# Brain functional network abnormalities in Parkinson’s disease patients at different disease stages

**DOI:** 10.3389/fnins.2025.1627838

**Published:** 2025-08-11

**Authors:** Wei Wei, Xinhui Wang, Chao Han, Yu Shen, Panlong Li, Yan Bai, Shuo Liu, Jingyao Xu, Yanhong Shi, Zhou Li, Meiyun Wang

**Affiliations:** ^1^Department of Radiology, Zhengzhou University People’s Hospital and Henan Provincial People’s Hospital, Zhengzhou, China; ^2^Department of Radiology, General Hospital of Pingmei Shenma Medical Group, Pingdingshan, China; ^3^Department of Radiology, Xinxiang Medical University and Henan Provincial People’s Hospital, Zhengzhou, China; ^4^Department of Neurology, General Hospital of Pingmei Shenma Medical Group, Pingdingshan, China

**Keywords:** Parkinson’s disease, resting-state functional MRI, brain functional networks, global topological organization, regional topological organization

## Abstract

**Background:**

Parkinson’s disease (PD) is a neurodegenerative disorder with some progressive impairment and an unclear pathogenesis.

**Purpose:**

This study aimed to use resting-state functional magnetic resonance imaging (rs-fMRI) and graph analysis approaches to compare changes in brain functional network topology in PD at different disease stages.

**Materials and methods:**

A total of 58 PD patients, comprising 29 early-stage PD (PD-E) and 29 middle-to-late stage PD (PD-M), and 29 age- and sex-matched healthy control (HC) participants, were recruited. All subjects underwent clinical assessments and magnetic resonance imaging (MRI) scanning. We analyzed alterations in the global, regional, and modular topological characteristics of brain functional networks among different disease stages of PD patients and HC participants. Furthermore, we also examined the relationship between topological features with significant group effects and clinical characteristics, including the Movement Disorder Society’s Unified Parkinson’s Disease Rating Scale III (MDS-UPDRS III) score and Hoehn and Yahr (H&Y) stage.

**Results:**

At the global level, PD-M and PD-E exhibited lower clustering coefficient, and PD-M also exhibited lower local efficiency and normalized characteristic path length relative to HC. At the regional level, PD-M and PD-E showed lower nodal centrality in temporal-occipital regions and higher centrality in brain regions related to the default mode network and the frontoparietal control network compared to HC. Notably, nodal centrality metrics of the left middle frontal gyrus and the temporal pole of the right middle temporal gyrus were associated with the MDS-UPDRS III score and H&Y stage.

**Conclusion:**

This study found that the brain functional networks were disrupted at varying degrees in patients with PD at different disease stages. These findings contribute to our understanding of the topological changes in the neural networks associated with the severity of PD.

## Introduction

Parkinson’s disease (PD) is a progressive neurological disorder characterized by motor symptoms, such as bradykinesia, rigidity, tremor, and postural instability, as well as non-motor features, including hyposmia, sleep disorders, depression, and constipation (Jankovic, 2008; Schapira et al., 2017). Its pathological process is mainly attributed to disruptions in the nigrostriatal dopamine system (Hayes, 2019). Currently, diagnosis and severity assessment of PD are still predominantly based on clinical symptoms (Gelb et al., 1999; Postuma et al., 2015). Developing reliable non-invasive biomarkers to monitor disease severity is important for future diagnosis and disease-modifying therapies in PD.

Since the 1990s, advancements in the physics of complex systems (Strogatz, 2001; Albert and Barabási, 2002; Boccaletti et al., 2006) and the rise of network science (Börner et al., 2008) have allowed us to study the structure and function of brain networks in terms of small-world topology, highly connected hubs, and modularity. In recent years, graph theory analysis has been widely employed in the study of complex brain networks (Bullmore and Sporns, 2009). By using graph theory analysis to study the interactions between neurons and the structure and function of neural networks, it is possible to better understand the information transmission between neurons and how neural networks operate, thereby making deeper contributions to the function of the nervous system and the mechanism of disease (Craddock et al., 2013). Neuroimaging has shown that PD is a neurodegenerative disorder involving many neurotransmitters, brain regions, structural and functional connections, and neurocognitive systems (Weingarten et al., 2015). Therefore, the integrated analysis of the whole brain networks may provide a more comprehensive understanding of brain abnormalities in PD.

Altered topological properties in functional networks have been reported in PD by several studies (Sang et al., 2015; Luo et al., 2015; Fang et al., 2017; Göttlich et al., 2013; Suo et al., 2017). The majority of these studies have focused on the alteration of brain functional networks in early-stage PD. For example, the study examining the topological configuration of brain networks in early-stage PD patients who received antiparkinson treatment found that global properties, module structure, and hub distribution were markedly altered in these patients (Sang et al., 2015). The results of these studies were inconsistent due to clinical heterogeneity (dopaminergic medication) among participants. Furthermore, these studies mainly focused on early-stage patients and were therefore unable to identify progressive brain changes across different stages. The configurations of the brain functional connectome in patients with PD were perturbed and correlated with disease severity (Suo et al., 2017). However, the topological properties of large-scale brain functional networks in patients with different stages of PD are unknown.

Our study aims to use resting-state functional magnetic resonance imaging (rs-fMRI) and graph analysis approaches to compare changes in brain functional network topology in PD at different disease stages.

## Methods and subjects

This study was conducted in accordance with the principles of the Declaration of Helsinki and was approved by the local human research ethics committee. Written informed consent was obtained from all participants (or their legal guardians) before enrollment.

### Subjects

All PD patients were continuously recruited from the Henan Provincial People’s Hospital from September 2020 to June 2023. We recruited 58 patients with a diagnosis of PD, as determined by two experienced neurologists, according to the clinical diagnostic criteria of the Movement Disorder Society (Postuma et al., 2015). All patients were assessed by the Movement Disorder Society’s Unified Parkinson’s Disease Rating Scale III (MDS-UPDRS III) (Goetz et al., 2008), the Hoehn and Yahr (H&Y) stage (Hoehn and Yahr, 1967), and the Mini-Mental State Examination (MMSE) (Folstein et al., 1983). Patients with H&Y ≤ 2.5 were assigned to the early-stage PD group (PD-E, *n* = 29), while those with H&Y ≥ 3 were assigned to the middle-to-late stage PD group (PD-M, *n* = 29) (Chen et al., 2016). The following patients were excluded: (1) parkinsonism syndrome and parkinsonism-plus syndrome (progressive supranuclear palsy, multiple system atrophy, and corticobasal degeneration); (2) patients who met general exclusion criteria for magnetic resonance imaging (MRI) scanning (such as those with claustrophobia or implanted metal parts); and (3) Individuals whose MMSE scores were lower than those corresponding to their educational level (The normal MMSE score is defined as follows: for illiterate individuals, it is >17; for those with 1–6 years of education, it is >20; for those with 7 years of education, it is >23) (Li et al., 2016). In addition, 29 age- and sex-matched healthy control (HC) participants who did not have any neurological disorders or structural brain defects were recruited.

### Data acquisition

All participants underwent an MRI examination using a 3-T system (MAGNETOM Prisma, Siemens Healthcare, Erlangen, Germany) equipped with a 64-channel head/neck coil. A foam pad was used to reduce head movement during scanning. All patients were taking antiparkinsonian drugs and were scanned while in the “on” state. MRI scanning parameters were as follows: for the structural T1-weighted sequence, repetition time (TR) of 2,300 ms, echo time (TE) of 2.28 ms, field of view (FOV) of 260 × 260 mm^2^, slice thickness of 1 mm, number of slices of 192, and voxel size of 1.0 × 1.0 × 1.0 mm^3^; for resting-state functional imaging, TR/TE of 2000/35 ms, FOV of 207 × 207 mm^2^, voxel size of 2.2 × 2.2 × 2.2 mm^3^, slice thickness of 2.2 mm, 75 axial slices, and 180 image volumes.

### Data processing

We used the graph theoretical network analysis (GRETNA) toolbox^1^ (Wang et al., 2015) to perform image preprocessing. Preprocessing steps were as follows: (1) DICOM to NIFTI; (2) removal of the first 10 time points; (3) slice timing corrections; (4) realignment to the mean volume for head motion correction, with exclusion of head motions > 3 mm and 3°; (5) spatial normalization using DARTEL segmentation; (6) removal of linear trends; (7) nuisance signal regression including 24 head motion parameters, cerebrospinal fluid (CSF), and white matter signals; (8) and band-pass filtering (0.01–0.08 Hz).

### Construction of brain functional networks

The network was also constructed using the GRETNA toolbox. A network consists of nodes and edges between nodes. The nodes represent brain regions, and the edges represent statistical interdependencies between blood oxygen level-dependent signals in different brain regions. To define network nodes, we used the automated anatomic labeling atlas to divide the entire brain into 90 cortical and subcortical regions of interest, each region representing a network node. The mean time series of each region is then obtained, and the Pearson’s correlations of the mean time series between all node pairs are calculated, i.e., the edges of the network. This generated a weighted 90 × 90 correlation matrix for each participant.

### Analysis of brain functional networks

To ensure the same number of edges among the three groups and to better observe the prominent small-world properties in the brain network, a wide range of sparsity was selected from 0.06 to 0.50 with a step of 0.01 in computing network metrics. An upper sparsity threshold of 0.5 was applied to preserve small-worldness (Sigma > 1.1), which is a fundamental topological property of functional brain networks. A lower threshold of 0.06 ensures that the network retains sufficient topological structure at lower sparsities, guarding against excessive sparsity and consequent information loss (Watts and Strogatz, 1998; Xie et al., 2025). We calculated the global and nodal network metrics for the brain networks at each sparsity level and the area under the curve over the sparsity range (Zhang et al., 2011). The global metrics were small-world parameters (Watts and Strogatz, 1998) and network efficiency parameters (Latora and Marchiori, 2001): clustering coefficient (Cp), characteristic path length (Lp), normalized clustering coefficient (Gamma), normalized characteristic path length (Lambda), and small worldness (Sigma), as well as global efficiency (Eglobal) and local efficiency (Elocal). The nodal centrality metrics were nodal degree, nodal efficiency, and nodal betweenness (Achard and Bullmore, 2007).

Based on previous literature (Suo et al., 2022), the 90 brain regions defined by the AAL90 atlas were categorized into five functional modules, and the intra- and inter-modular connectivity among these modules was analyzed. The five modules consisted of (I) the sensorimotor module, (II) the default mode module, (III) the frontal–parietal module, (IV) the subcortical module, and (V) the visual module (Table 1).

**Table 1:** Modular organization.

Modules	Regions
Module I (sensorimotor module)	Bilateral precentral and postcentral gyrus, supplementary motor area, Rolandic operculum, paracentral lobule, insula, supramarginal gyrus, superior temporal gyrus, Heschl gyrus, and temporal pole: superior temporal gyrus
Module II (default mode module)	Bilateral superior frontal gyrus (dorsolateral, medial, orbital, and medial orbital part), rectus gyrus, olfactory cortex, cingulate gyrus (anterior, median, and posterior), angular gyrus, precuneus, inferior and middle temporal gyrus, and temporal pole: middle temporal gyrus
Module III (frontal–parietal module)	Bilateral inferior frontal gyrus (opercular, triangular, and orbital part), middle frontal gyrus, middle frontal gyrus (orbital part), and superior and inferior parietal gyrus
Module IV (subcortical module)	Bilateral hippocampus, parahippocampal gyrus, amygdala, caudate, putamen, pallidum, and thalamus
Module V (visual module)	Bilateral calcarine fissure and surrounding cortex, superior, middle and inferior occipital gyrus, lingual gyrus, cuneus, and fusiform gyrus

### Statistical analysis

The analyses of demographic and clinical data were performed using SPSS (Version 27.0; IBM) software, and *p* < 0.05 was considered statistically significant. Two-samples t-test, Mann–Whitney U-test, Kruskal–Wallis H test, and chi-squared test were performed to compare quantitative and qualitative variables. We used the GRETNA statistics modules for the statistical analysis of the area under the curve (AUC) values of network metrics. Analysis of covariance (the node metrics need to be false discovery rate correction corrected with a significance threshold of *q* < 0.05) with age, gender, and education scores as covariates to determine network differences among the three groups. We extracted the values of the areas under the curve of topological attributes for each region with significant changes and subsequently compared patients at different stages and HC participants using a *post hoc* two-samples t-test (*p* < 0.05, Bonferroni-corrected). Finally, partial correlations were computed to examine relationships between these values and the UPDRS III score and H&Y stage in PD, with age, gender, and education score as covariates.

## Results

### Demographic and clinical characteristics

Demographic and clinical characteristics of 58 patients with PD and 29 HC participants are shown in Table 2. There were no significant differences in gender or years of education among the PD-E, PD-M, and HC groups (*p* > 0.05). Additionally, the MMSE scores and age at onset did not significantly differ between the PD-E and PD-M groups (*p* > 0.05). The age of patients with PD-M was higher than that of PD-E and HC participants (*p* = 0.013 and *p* = 0.011). PD-E and PD-M patients had statistically significant differences in disease duration (*p* = 0.004) and the UPDRS-III score (*p* < 0.001).

**Table 2:** Demographics and clinical characteristics of PD-E and PD-M patients and HC participants.

Parameter	Groups			*p*	Post hoc *p*-value		
	PD-E	PD-M	HC		HC vs. PD-E	HC vs. PD-M	PD-E vs. PD-M
Age (y)	62.00 (54.00,65.50)	65.00 (58.50,70.50)	62.00 (54.50,66.00)	**0.015**	0.942	**0.011**	**0.013**
Gender (female/male)	12/17	14/15	13/16	0.870	NA	NA	NA
Education (y)	9 (6,12)	9 (6,12)	9 (9,12)	0.477	NA	NA	NA
Disease duration (y)	3.0 (1.5,5.0)	5.0 (3.0,9.0)	NA	**0.004**	NA	NA	NA
MDS-UPDRS III	26.21 ± 12.11	54.00 ± 17.93	NA	**<0.001**	NA	NA	NA
MMSE	25.00 (19.50,28.00)	25.00 (23.00,27.00)	NA	0.790	NA	NA	NA
Age at onset (y)	56.90 ± 5.54	59.72 ± 8.54	NA	0.141	NA	NA	NA

Data are expressed as mean ± standard deviation for normally distributed data and as median (25th, 75th percentiles) for non-normally distributed data. PD-E, early-stage Parkinson’s disease; PD-M, middle-to-late stage Parkinson’s disease; HC, healthy control; MDS-UPDRS III, Movement Disorder Society’s Unified Parkinson’s Disease Rating Scale III; MMSE, Mini-Mental State Examination, NA, not applicable. Bold *p*-values indicate statistically significant group effects (*p* < 0.05).

### Global topological organization of brain functional networks

**Figure 1:**
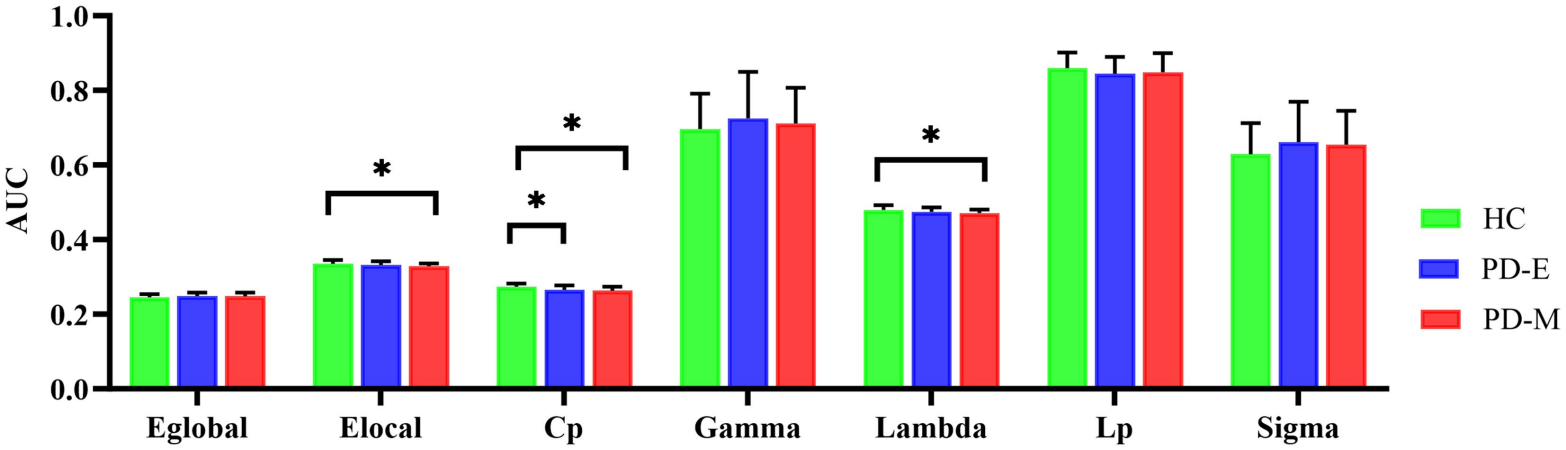
Differences in global topological organization of the functional brain network among the three groups (*p* < 0.05). PD-E, early-stage Parkinson’s disease; PD-M, middle-to-late stage Parkinson’s disease; HC, healthy control; AUC, area under the curve; Eglobal, global efficiency; Elocal, local efficiency; Cp, clustering coefficient; Lp, characteristic path length; Gamma, normalized clustering coefficient; Lambda, normalized characteristic path length; Sigma, small worldness.

Significant group effects were found in the AUCs of Cp, Elocal, and Lambda (*p* < 0.001, *p* = 0.036, and *p* = 0.029, respectively). *Post hoc* testing showed that compared to HC, PD-M patients had significantly lower Cp (*p* < 0.001), Elocal (*p* = 0.010), and Lambda (*p* = 0.009), while PD-E patients had significantly lower Cp (*p* = 0.004). However, there is no significant difference in global metrics between PD-E and PD-M patients. No significant difference was identified in Eglobal, Lp, Gamma, and Sigma values (Table 3 and Figure 1).

**Table 3:** Brain network metrics differences among the PD-E and PD-M patients and HC participants.

Measurements	Groups			*p*	Post hoc *p*-value		
	PD-E	PD-M	HC		HC vs. PD-E	HC vs. PD-M	PD-E vs. PD-M
Global							
Eglobal	0.249 ± 0.009	0.248 ± 0.010	0.245 ± 0.009	0.290	NA	NA	NA
Elocal	0.332 ± 0.010	0.329 ± 0.007	0.335 ± 0.010	**0.036**	0.146	**0.010**	0.248
Cp	0.265 ± 0.012	0.263 ± 0.011	0.273 ± 0.009	**<0.001**	**0.004**	**<0.001**	0.409
Lp	0.845 ± 0.045	0.849 ± 0.050	0.859 ± 0.042	0.457	NA	NA	NA
Gamma	0.726 ± 0.124	0.711 ± 0.096	0.696 ± 0.095	0.567	NA	NA	NA
Lambda	0.474 ± 0.012	0.471 ± 0.009	0.479 ± 0.013	**0.029**	0.112	**0.009**	0.279
Sigma	0.661 ± 0.109	0.654 ± 0.091	0.629 ± 0.084	0.391	NA	NA	NA
Nodal degree							
MFG.L	13.048 ± 3.583	16.298 ± 3.544	12.751 ± 4.460	**0.001**	0.771	**0.002**	**0.006**
ORBmid.R	8.701 ± 3.264	11.785 ± 4.288	7.969 ± 3.560	**<0.001**	0.457	**<0.001**	**0.007**
FFG.L	13.566 ± 3.696	13.502 ± 4.027	16.719 ± 3.497	**0.002**	**0.006**	**0.005**	0.949
FFG.R	14.920 ± 3.073	12.547 ± 3.863	15.571 ± 2.890	**0.002**	0.455	**0.002**	**0.023**
ANG.L	9.515 ± 4.394	10.846 ± 3.519	6.902 ± 3.667	**<0.001**	**0.036**	**<0.001**	0.195
TPOmid.L	6.885 ± 3.771	6.739 ± 3.578	10.535 ± 4.187	**<0.001**	**0.002**	**<0.001**	0.886
Nodal efficiency							
MFG.L	0.272 ± 0.023	0.293 ± 0.022	0.267 ± 0.312	**<0.001**	0.470	**<0.001**	**0.008**
ORBmid.R	0.238 ± 0.032	0.262 ± 0.031	0.229 ± 0.034	**<0.001**	0.298	**<0.001**	**0.017**
SFGmed.L	0.271 ± 0.023	0.281 ± 0.022	0.256 ± 0.037	**0.005**	0.051	**0.004**	0.171
PCG.R	0.223 ± 0.462	0.223 ± 0.046	0.178 ± 0.073	**0.003**	**0.010**	**0.008**	0.962
FFG.L	0.273 ± 0.029	0.271 ± 0.031	0.293 ± 0.022	**0.005**	**0.019**	**0.010**	0.833
FFG.R	0.282 ± 0.021	0.263 ± 0.030	0.286 ± 0.019	**<0.001**	0.610	**0.002**	**0.008**
ANG.L	0.241 ± 0.040	0.256 ± 0.027	0.218 ± 0.037	**<0.001**	**0.035**	**<0.001**	0.129
ANG.R	0.248 ± 0.035	0.258 ± 0.022	0.228 ± 0.038	**0.003**	0.054	**0.002**	0.279
TPOmid.L	0.214 ± 0.049	0.217 ± 0.038	0.248 ± 0.037	**0.004**	**0.008**	**0.016**	0.795

Data are mean ± standard deviations. PD-E, early-stage Parkinson’s disease; PD-M, middle-to-late stage Parkinson’s disease; HC, healthy control; Eglobal, global efficiency; Elocal, local efficiency; Cp, clustering coefficient; Lp, characteristic path length; Gamma, normalized clustering coefficient; Lambda, normalized characteristic path length; Sigma, small worldness; MFG.L, left middle frontal gyrus; ORBmid.R, orbital part of the right middle frontal gyrus; SFGmed.L, left superior frontal gyrus; PCG.R, right posterior cingulate gyrus; FFG.L, left fusiform gyrus; FFG.R, right fusiform gyrus; ANG.L, left angular gyrus; ANG.R, right angular gyrus; TPOmid.L, temporal pole of the left middle temporal gyrus; NA, not applicable. Bold values indicate statistically significant group effects (*p* < 0.05 for global metrics; *q* < 0.05 after FDR correction for nodal metrics, with post-hoc pairwise comparisons Bonferroni-corrected at *p* < 0.05).

### Regional topological organization of brain functional networks

We identified the brain regions that showed significant between-group differences in at least one nodal metric (*q* < 0.05, false discovery rate corrected). Significant group differences were revealed in the left middle frontal gyrus (MFG.L), orbital part of the right middle frontal gyrus (ORBmid.R), medial part of the left superior frontal gyrus (SFGmed.L), right posterior cingulate gyrus (PCG.R), left fusiform gyrus (FFG.L), right fusiform gyrus (FFG.R), left angular gyrus (ANG.L), right angular gyrus (ANG.R), and temporal pole of the left middle temporal gyrus (TPOmid.L) (Table 3).

**Figure 2:**
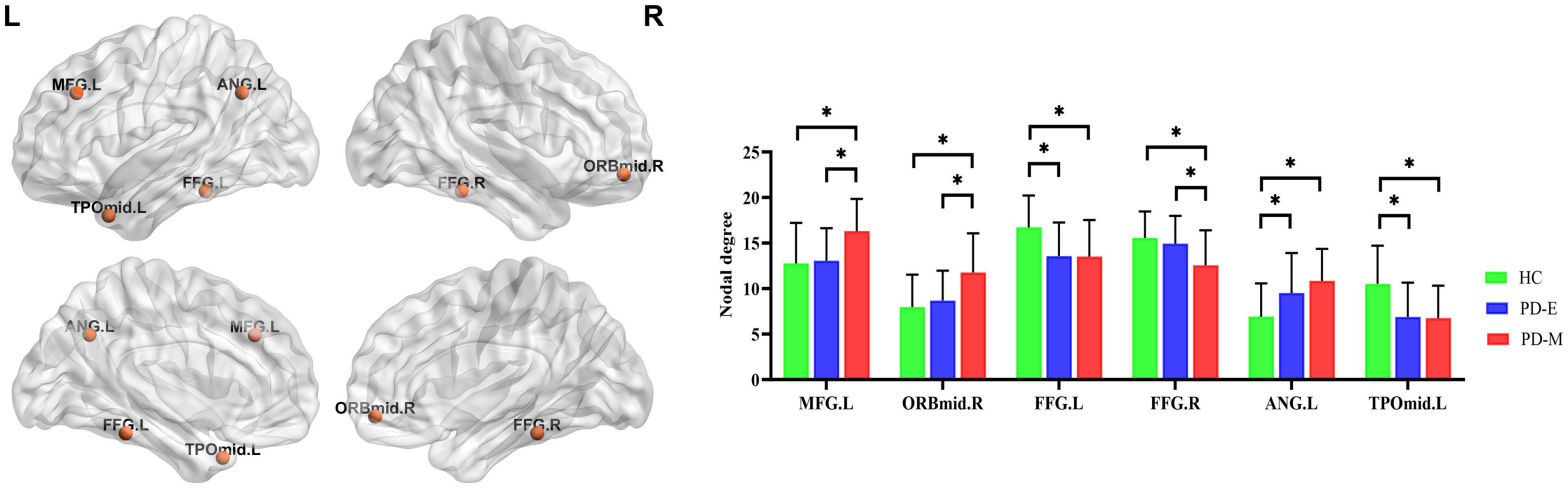
Nodes with significant differences in the nodal degree among the three groups (*q* < 0.05, false discovery rate corrected). PD-E, early-stage Parkinson’s disease; PD-M, middle-to-late stage Parkinson’s disease; HC, healthy control; MFG.L, left middle frontal gyrus; ORBmid.R, orbital part of the right middle frontal gyrus; FFG.L, left fusiform gyrus; FFG.R, right fusiform gyrus; ANG.L, left angular gyrus; TPOmid.L, temporal pole of the left middle temporal gyrus; L, left; R, right.

Compared to HC, PD-M patients showed significantly decreased nodal degrees in the FFG.L (*p* = 0.005), FFG.R (*p* = 0.002), and TPOmid.L (*p* < 0.001), with increased values in the MFG.L (*p* = 0.002), ORBmid.R (*p* < 0.001), and ANG.L (*p* < 0.001). PD-E patients exhibited decreased nodal degree in the FFG.L (*p* = 0.006) and TPOmid.L (*p* = 0.002), along with increased values in the ANG.L (*p* = 0.036) compared to HC. PD-M patients demonstrated lower nodal degree in the FFG.R (*p* = 0.023) but higher values in the MFG.L (*p* = 0.006) and ORBmid.R (*p* = 0.007) compared to PD-E patients (Figure 2).

**Figure 3:**
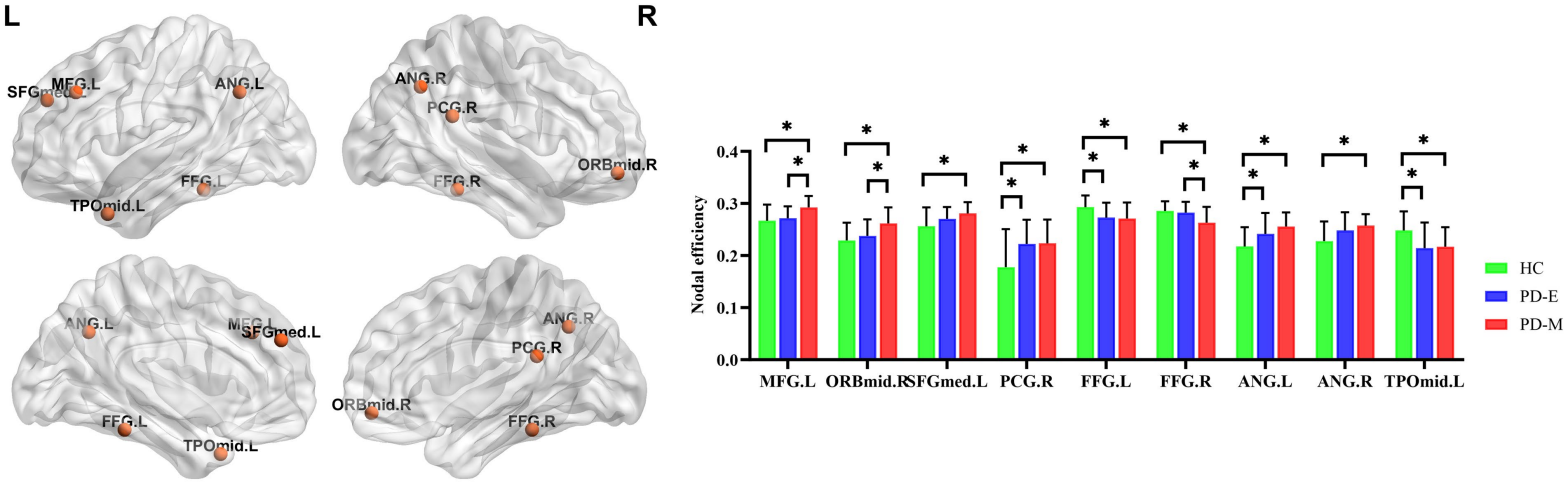
Nodes with significant differences in nodal efficiency among the three groups (*q* < 0.05, false discovery rate corrected). PD-E, early-stage Parkinson’s disease; PD-M, middle-to-late stage Parkinson’s disease; HC, healthy control; MFG.L, left middle frontal gyrus; ORBmid.R, orbital part of the right middle frontal gyrus; SFGmed.L, left superior frontal gyrus; PCG.R, right posterior cingulate gyrus; FFG.L, left fusiform gyrus; FFG.R, right fusiform gyrus; ANG.L, left angular gyrus; ANG.R, right angular gyrus; TPOmid.L, temporal pole of the left middle temporal gyrus; L, left; R, right.

Compared to HC, PD-M patients showed significantly decreased nodal efficiency in the FFG.L (*p* = 0.010), FFG.R (*p* = 0.002), and TPOmid.L (*p* = 0.016), with increased values in the MFG.L (*p* < 0.001), ORBmid.R (*p* < 0.001), SFGmed.L (*p* = 0.004), PCG.R (*p* = 0.008), ANG.L (*p* < 0.001), and ANG.R (*p* = 0.002). PD-E patients exhibited decreased nodal efficiency in the FFG.L (*p* = 0.019) and TPOmid.L (*p* = 0.008), along with increased values in the PCG.R (*p* = 0.010) and ANG.L (*p* = 0.035) compared to HC. PD-M patients demonstrated lower nodal efficiency in the FFG.R (*p* = 0.008) but higher values in the MFG.L (*p* = 0.008) and ORBmid.R (*p* = 0.017) compared to PD-E patients (Figure 3).

### Modular interactions of brain functional networks

**Figure 4:**
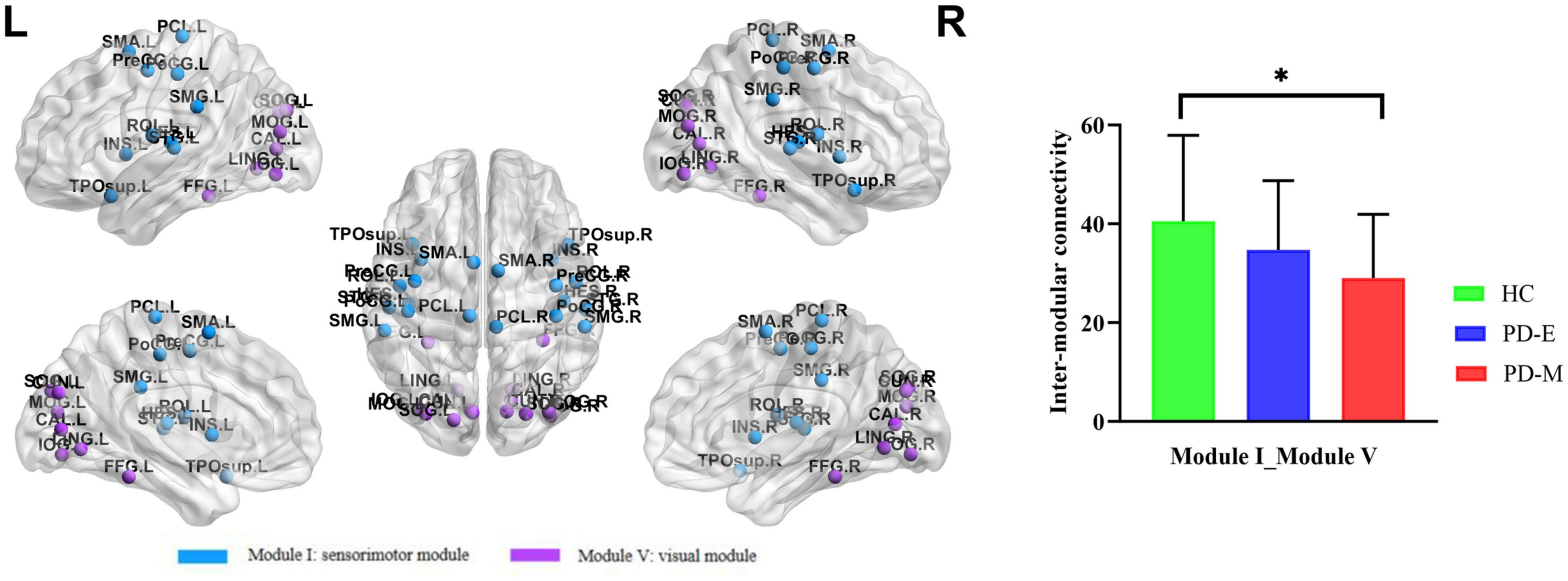
Modular interaction with differences among the three groups (*p* < 0.05, uncorrected). PD-E, early-stage Parkinson’s disease; PD-M, middle-to-late stage Parkinson’s disease; HC, healthy control; L, left; R, right.

No significant differences in intra- or inter-modular connectivity were observed between groups in corrected analyses. However, in uncorrected analyses, PD-M patients exhibited reduced functional connectivity between Module I and Module V compared to healthy controls (*p* = 0.017, uncorrected) (Figure 4).

### Correlation analysis

**Figure 5:**
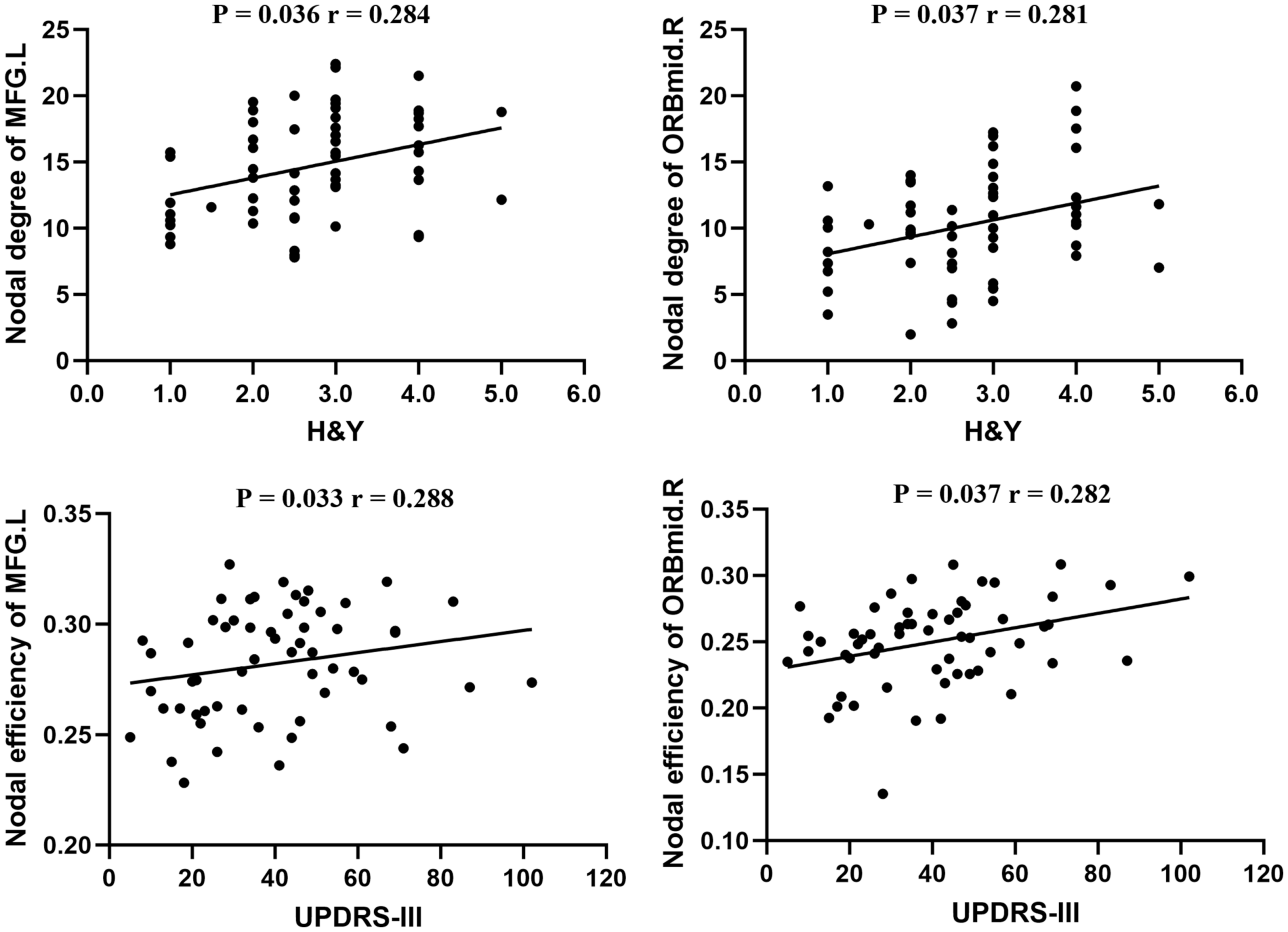
Relationship between nodal metrics and the severity of clinical diseases in Parkinson’s disease (*p* < 0.05). MFG.L, left middle frontal gyrus; ORBmid.R, orbital part of the right middle frontal gyrus; H&Y, Hoehn and Yahr; MDS-UPDRS III, Movement Disorder Society’s Unified Parkinson’s Disease Rating Scale III.

We also examined the relationships between nodal metrics with significant group effects and clinical characteristics (UPDRS-III score and H&Y stage), with age, gender, and education scores as covariates. In patients with PD, nodal degree of MFG.L (*p* = 0.036, *r* = 0.284) and ORBmid.R (*p* = 0.037, *r* = 0.281) was positively correlated with H&Y stage. The nodal efficiency of MFG.L (*p* = 0.033, *r* = 0.288) and ORBmid.R (*p* = 0.037, *r* = 0.282) were positively correlated with the UPDRS-III score (Figure 5).

## Discussion

In this study, we investigated the topological properties of brain functional networks in PD patients at different disease stages and HC. Patients with PD showed abnormalities at both the global level (decreases in the Cp, Elocal, and Lambda) and the nodal level (decreased nodal centrality metrics in the temporal-occipital regions, but increased in brain regions related to the default mode network and the frontoparietal control network). It is worth noting that patients with PD-M exhibit more extensive changes in topological attributes compared to those with PD-E. Furthermore, nodal centrality metrics of the MFG.L and ORBmid.R were positively correlated with the H&Y stage and UPDRS-III score. These findings may enhance our understanding of the mechanisms underlying the progression of PD and contribute to the development of non-invasive neuroimaging biomarkers for monitoring disease progression.

Networks with low construction costs but high efficiency in disseminating information are called economic small-world networks. Since the combination of high local clustering and short path length supports the two fundamental organization principles of functional segregation and functional integration in the brain, the small-world is an attractive model to describe brain networks (Zhang et al., 2011). In our study, the networks in PD patients were perturbed in a way that may reflect the underlying pathophysiologic abnormalities and disease progression. Regarding global topologic properties, Cp and Elocal were significantly decreased, whereas Lp and Eglobal were not significantly different in PD-M patients compared to HC. Since both Cp and Elocal measure local cliquishness of the network, and Lp and Eglobal are the most commonly used measures of functional integration (Latora and Marchiori, 2001), our results indicate a disturbance in the normal balance in functional brain networks of PD-M patients. These results are consistent with one functional connectome study, which reported decreased network segregation in drug-naive PD with no significant change in network integration (Luo et al., 2015). However, other studies (Suo et al., 2017; Ma et al., 2017) reported decreased local specialization and global integration in early-to-mid-stage PD and a progressive impairment in local specialization with an additional loss of global integration in PD. In accordance with reports, dopaminergic antagonists can reduce both global and local efficiency in healthy subjects (Achard and Bullmore, 2007), while dopamine-based medications can increase the functional connectivity between dopamine-related cognitive and motor pathways in healthy individuals (Kelly et al., 2007). Subsequent studies in PD patients have found that dopaminergic medication is also considered to partially restore the deficits in brain functional networks in PD patients (Delaveau et al., 2010; Palmer et al., 2010). In the above study, medication was withdrawn at least 12 h (off-state) in PD patients before resting-state functional MR imaging, and we were in the on-state, so we hypothesized that medication might contribute to the difference. In addition, PD-E patients only had lower Cp than HC participants in our study, which might be attributed to the effective symptom management of PD-E patients following medication. Future research could conduct medication-specific subgroup analyses, such as comparing medicated and unmedicated patients, to further elucidate the impact of pharmacological interventions on these findings.

In addition to these altered global topologic properties, PD patients at different disease stages have selectively and significantly impaired nodal centrality metrics in several regions of the brain’s functional networks, mainly including the decreased temporal-occipital regions and the increased brain regions related to the default mode network and the frontoparietal control network. Abnormalities of gray matter (Goldman et al., 2014), neuronal activity (Meppelink et al., 2009), and nodal centrality metrics (Luo et al., 2015) in temporal-occipital regions have been reported in PD patients compared to HC. The temporal-occipital regions are important for visual object recognition, so these changes are thought to be related to abnormal bottom-up visual processing of visual information in these regions and visual-cognitive deficits in PD patients (Meppelink et al., 2009). We found that PD-E patients had decreased nodal centrality metrics in the temporo-occipital brain regions, and the abnormalities were more obvious in PD-M patients. This finding is consistent with previous findings that have shown PD patients exhibit functional changes in the cortical visual system before visual symptoms are clinically evident (Cardoso et al., 2010; Uc et al., 2005). The default mode network and frontoparietal control network serve as core neural networks for cognitive control (Menon and D'Esposito, 2021). Previous research has indicated that even patients with PD-E, who have not yet been formally diagnosed with mild cognitive impairment, may experience subtle cognitive declines (Kandiah et al., 2009; Aarsland et al., 2017). Interestingly, although our study’s PD patients did not exhibit overt cognitive deficits, we observed significantly increased nodal degree and nodal efficiency within brain regions associated with the default mode network and the frontoparietal control network at different disease stages. This finding may reflect a potential adaptive response, characterized by increased connectivity and efficiency, which could support cognitive stability. However, further validation through targeted neuropsychological assessments is required to confirm compensatory processes in future studies.

The middle frontal gyrus is a key part of the prefrontal network that responds to inhibit responses to inappropriate stimuli by overriding the motor system’s automatic response tendency, and one study has shown that with age and the development of behavioral control, the specialization and organization of the response inhibition network improve. In contrast, the activation of the right middle frontal gyrus decreases significantly (Hardee et al., 2014). Inhibition control is an important executive function that involves inhibiting dominant responses or allowing appropriate actions to meet complex task requirements and adapt to different environments (Li and Sinha, 2007). A recent study on impulse control disorders in PD has shown that PD patients with impulse control disorders show reduced voxel-mirrored homotopic connectivity (VMHC) values in the MFG, middle and superior orbital frontal gyrus, inferior frontal gyrus, and caudate, which can detect altered interhemispheric connectivity by quantifying functional connections between the corresponding voxels in the two hemispheres of the brain (Gan et al., 2021). Therefore, this result suggested that the bilateral connections between the cerebral hemispheres of PD patients with impulse control disorders are altered, and the severity of impulse control disorders is correlated with the mean VMHC values of MFG. Our research findings indicate that as the H&Y stage and UPDRS-III scores advance in patients with PD, there is a corresponding increase in the nodal degree and nodal efficiency of the MFG.L and ORBmid.R. This result may complement previous studies suggesting that PD patients may compensate for the weak anatomical connections between the inhibited areas by increasing executive control.

Additionally, in module analysis, reduced functional connectivity was observed between the sensorimotor and visual modules in PD-M patients. Sensorimotor network plays a crucial role in integrating sensory inputs and facilitating motor execution, whereas the visual network contributes to spatial orientation and the processing of visual feedback (Bertoni et al., 2025; Kim et al., 2024). Previous research has shown that the sensorimotor network in PD patients is unable to effectively integrate feedback from the visual network, leading to motor dysfunction (Caspers et al., 2021). However, the results of our study did not survive multiple comparison correction and warrant further investigation to validate its clinical relevance.

There are several limitations to our study. First, the sample size was relatively small, which may have constrained our ability to detect the expected sensorimotor network impairments consistent with typical motor manifestations of PD. In the future, the sample size will be increased to continue the research. Second, the observation of reduced functional connectivity between sensorimotor and visual modules in PD-M patients did not survive multiple comparison correction and should be considered exploratory. Larger and independent datasets are needed to validate these findings, alongside studies using advanced neuroimaging approaches (e.g., task-based fMRI) to uncover their functional roles and biological foundations. Finally, all rs-fMRI data were acquired during the dopaminergic “ON” state to optimize patient comfort and scan quality. While clinically practical, this approach precludes the dissociation between medication effects and intrinsic PD-related network pathology. Previous studies demonstrate that dopaminergic agents can partially normalize aberrant functional connectivity patterns in PD patients (Delaveau et al., 2010; Palmer et al., 2010). Consequently, future studies should implement counterbalanced ON/OFF state designs with standardized medication withdrawal protocols. Additionally, the potential dose-dependent effects of levodopa equivalent daily dose (LEDD) on functional network topology were not examined due to incomplete pharmacological records. Future studies should incorporate stratified LEDD cohorts to resolve this critical confounding.

## Conclusion

In conclusion, we used rs-fMRI and graph analysis approaches to compare changes in brain functional network topology in PD at different disease stages. We observed that patients with PD-M exhibited a reduction in Cp, Elocal, and Lambda. Additionally, both PD-E and PD-M patients demonstrated decreased nodal centrality metrics within the temporal-occipital regions, alongside an enhanced brain region related to the default mode network and frontoparietal control network. Notably, patients with PD-M exhibit more extensive changes in topological attributes compared to those with PD-E. Furthermore, nodal centrality metrics of the MFG.L and ORBmid.R were positively correlated with the H&Y stage and UPDRS-III score. These findings contribute to our understanding of the topological changes in the neural networks associated with the severity of PD.

## Data Availability

The datasets generated and analyzed during the current study are not publicly available due to patient privacy concerns. De-identified data may be made available to qualified researchers upon reasonable request, subject to approval by the local human research Ethics Committee. Requests should be directed to mywang@zzu.edu.cn.

## Footnotes

^1^ http://www.nitrc.org/projects/gretna/
